# Evaluation of Patients with Painful Bladder Syndrome/Interstitial Cystitis

**DOI:** 10.1100/tsw.2005.119

**Published:** 2005-12-01

**Authors:** Jean Jacques Wyndaele

**Affiliations:** Department of Urology, University Antwerp, 10 Wilrijkstraat, B 2650 Edegem, Belgium

**Keywords:** interstitial cystitis, painful bladder syndrome, diagnosis, chronic pelvic pain syndrome, pain

## Abstract

This review looks into the evaluation of patients with interstitial cystitis (IC). Interstitial cystitis is not easy to define. There is a lot of activity in this domain and a great international effort is made to get to a generally accepted definition and standardised protocols for diagnosis and treatment. We have not reached this point so far.

The diagnosis is still one of exclusion but search for specific markers may change this. Till then other conditions have to be looked for and the list of these is not short. A primary evaluation with history taking, symptom scores, clinical investigations, urine test can guide the physician to further testing.

Urodynamic tests, cystoscopy and hydrodistension, biopsies and pathologic evaluation are in the process of finding a place in the diagnostic tree. The potassium test seems useful for the evaluation of bladder permeability but is not specific for IC alone.

Studies on reevaluation in patients with IC show that primary misdiagnosis happens in a number of them. This is an extra motivation to continue the work towards standardization of the diagnostic and therapeutic approach.

### INTRODUCTION

Much has been going on in recent years in the domain of interstitial cystitis (IC) and other pain disorders of the pelvic region. This evolution is good because historically there has been much confusion about IC and patients have received lesser care for a long time.

IC is not easy to define and it is not unlikely that several conditions are to be found under the same denominator.

In 1988, NIDDK published criteria for IC[1] in an effort to make comparisons between possible study materials. In 2003, an international consultation in Kyoto, Japan started the process to update a proper definition and to obtain consensus on diagnosis and treatment modalities. The results were published in a special issue of the *International Journal of Urology* (Vol. 10, supplement in 2003). This was followed by international meetings in Alexandria (Virginia), Copenhagen, Rome, Baden, and several others. The 3rd International Consultation on Incontinence devoted a special working group on Painful Bladder Syndrome, from which the results have been published in 2005[2]. Different societies have been created to continue the study of IC: the European Society for the Study of IC (ESSIC), the Multinational Interstitial Cystitis Association (MICA), etc. Influential patient groups have developed in many countries in the world.

This manuscript gives a review of what the latest proposals are for the evaluation of patients with IC.

### DEFINITION

The ICI committee decided to follow the standard terminology of the International Continence Society (ICS). Painful bladder syndrome (PBS) is preferred to IC and is defined as “the complaint of suprapubic pain related to bladder filling, accompanied by other symptoms as daytime and night time frequency in the absence of proven urinary infection or other obvious pathology”. ICS describes IC as a specific diagnosis that requires confirmation by typical cystoscopic and histological features[3]. There is no general consensus on what such criteria should be. The ICI committee 2005 leaves the screening to exclude other relevant diagnoses and a focused evaluation at the discretion of the physician or center.

PBS is one urological condition of the group of chronic pelvic pains, nonmalignant pain conditions perceived in structures related to the pelvis. There seems to be general agreement that pain must be present in order to consider the possible diagnosis of IC[4]. Many patients, however, have only one symptom at onset of the disease[5] and most receive the initial diagnosis of cystitis. A condition of recurrent bacterial urinary tract infections with subsequent persistence of symptoms and negative cultures could be detected as a harbinger of IC in 60% of the patients.

### DIAGNOSIS

The primary task is to exclude an impressive list of diseases that can cause similar symptoms and signs. Infections, malignancies, lower urinary tract dysfunctions, gynecological and anorectal diseases, systemic diseases, neuropathies, stones and obstructions, urethral diverticula, prostatitis of type II and III A, muscular problems, and diseases of the joints are theoretically to be searched for and to be excluded.

How far one must go in doing this has not been well defined as yet. Many publications are not free of some confusion between this primary diagnostic workout with exclusion of other diseases and the specific evaluation if IC has been accepted as the cause of the complaints. However, it would seem that the best approach will be a standard basic workup and extending the diagnostic testing from the subsequent information gathered.

Primary evaluation of IC patients has been well defined by the Copenhagen Consensus meeting organised by Nordling et al. in 2004[6].

In the beginning, the initial diagnostic workup will not differ from what is done at most medical consultations. It will start with a specific and general medical history including the symptoms and signs that bring the patient to seek help. Pain is an essential element of IC. Pain should be located at bladder level and will be related by many patients to bladder filling. Though the diagnosis of IC is, to a large extent, made by exclusion, there are a few important positive factors. Pain increasing with bladder filling, relieved by voiding, only to reappear after some time when the bladder fills again is such a factor. However, pain may not get better or disappear after voiding in all patients. Pain can also be localized in the urethra, vagina, perineum, deep in the lower abdomen, lower back, rectum, or elsewhere (Fitzgerald et al. ICDB Study group ICS/IUGA congres Paris 2004, abstract 417). The pain can have all types, i.e., from sharp to dull, from burning to itching, or stabbing.

A big problem in many chronic patients is that the specificity of pain seems to get lost and all sensations elicited in the pelvic and genital area may get involved.

Other symptoms apart from the pain can be the result of shrinking bladder capacity, overall increase of sensation, and fear from pain; frequent voiding, awakening at night to void, urgent voids, and urgency can also be present. Dyspareunia can be present. Pain can flare with ovulation, menses, or before menses. The symptoms may be continuous or undulating. Emphasis should go to previous surgery and infections, urological diseases, radiation therapy, autoimmune diseases, drug intake, diabetes, tuberculosis, and more.

It is well known that symptoms are not very reliable in many medical conditions such as incontinence and overactive bladder[7,8]. The transfer of information has a rather complicated sequence: observation of the symptoms by the patient, transforming this information into words, verbally transferring them to the doctor, receipt of this information and interpretation of the data by the doctor; the traject is rather hazardous. When IC patients relate their history, clinical experience shows that it is no better than what has been described in incontinence and other conditions. The need exists to make spontaneous reporting and the information received from questioning more trustworthy.

It has become evident that the NIDDK criteria mandate underdiagnosis and deny treatment to many patients[9]. The evolution goes towards a broader acceptance of diagnostic criteria.

### Voiding Diary (VD)

Voiding diaries can help to make the information about voiding habits more clear and objective. Information, clear instructions, and encouragement are important for all types of VD. It is difficult to propose one type for all patients. Adaptation of the VD to the data needed and to the patient’s possibilities seems more logical. More complex diaries can be used for research and are probably not even ideal for clinical trails.

Volume and number of fluid intake and output should be written down for 3 days (day and night) as this permits a good general impression and an evaluation of consistency of the data. If sensation is also recorded, the information will be even more valuable and related to urodynamic sensory data[10]. Highest volumes with and without holding maneuvers can be used to estimate functional capacity.

### Symptom Scores

Symptoms scores are useful, such as the O’Leary-Sant Interstitial Cystitis Symptom and Problem Index (ICSI and ICPI)[11] supplemented with a suitable sex score with a quality of life score. The Copenhagen group advocates the IPSS Quality of Life score[6]. Another scoring is the University of Wisconsin Symptom Instrument (U-W). The U-W scoring has been validated again recently[12]. The study showed that IC patients had significantly higher scores for the seven urinary symptoms compared to controls. The duration of the disease correlated with the symptoms. The U-W was also recently compared with the ICSI in 107 patients[13]. A strong correlation was found between the two.

Pain can be recorded using a Visual Analogue Scale and can be advocated for the average, mildest, and worst pain during the last 24 h[6].

### Physical Examination

This has to be done as a general evaluation with inspection and palpation of the whole area while trying to localize the most exact location of the pain. Patients can help by telling in which position, with which leg movements, etc. the pain exacerbates. Such is not always possible as patients can complain of pain that is not to be elicited during the examination. Pain mapping is advocated. Vaginal and rectal palpation also permit evaluation of the pelvic floor muscles. Basic neurological testing allows us to exclude gross deficits in the lumbosacral and perineal innervation.

### Urine Tests

- Tests on urine have to be done to exclude infection, hematuria, and sterile pyuria.
- Cytology can be done in risk groups.
- Tests for Ureaplasma and Chlamydia can be done.

At this stage, it will already be possible to exclude several pathologies as infection, gross anatomical deficits, or to get indications for further tests.

#### Potassium Test

The test developed by Parsons[14] assesses sensitivity to intravesical instillation of potassium. The classic test elicits responses to 40 ml of plain water or 40 ml of potassium chloride solution, 40 mEq per 100 cc of water. The subjects are asked to rate their subjective responses on a scale of 0 to 5 (corresponding to no change from baseline to severe pain) 3–5 min after instillation of each solution and to compare the two. The test is negative as neither water nor potassium provokes symptoms. A positive test shows no reaction to water and pain or urgency after potassium. This may indicate increased permeability of the urothelium to potassium, increased acuity of response of the bladder nerves, or a combination of the two. After a full evaluation of the literature, the ICI committee[2] concluded that the test cannot be recommended for general use as a diagnostic tool for IC as neither a high sensitivity or specificity has been established. Although a significant increase in pain can occur with instillation of KCl in patients with IC, the scores and the rate of positive tests is not found different from those in healthy subjects[15].

A modified KCl test has been proposed by Daha et al.[16]. A Foley catheter is inserted and the bladder emptied. Then the bladder is filled at 50 ml/min until capacity. This volume is measured. The instillation is repeated with a solution of 0.2 *M* KCl at 50 ml/min until maximal capacity again. If a capacity drop of greater than 30% is found, the test is considered positive. With KCl, there is a stronger feeling of urgency in IC patients compared with the saline filling.

#### Biomarkers

Many biomarkers have been studied. Antiproliferative Factor is emerging as the best candidate for a biomarker for symptomatic IC. Also heparine-binding epidermal growth factor-like growth factor (HB-EGF), epidermal growth factor (EGF), insulin-like growth factor 1 (IGF1), and insulin–like growth factor binding protein 3 (IGFBP3) have been shown to be significantly different between IC patients, asymptomatic patients, and patients with acute bacterial cystitis[17]. Possible markers for IC severity may be urinary urinate and sulfated glycosaminoglycan levels[18].

A new approach may be the use of proteomic and metabonomic patterns for the diagnosis of IC. The methodology relies on advanced bioinformatics to scrutinize information contained within mass spectrometry and high-resolution proton nuclear magnetic resonance spectral patterns. In a recent study, Van et al.[19] were able to discriminate spectral patterns associated with IC affected, bacterial cystitis affected, and unaffected patients with a success rate of approximately 84%.

#### Urodynamic Tests

It is doubtful that routine urodynamics should be mandatory in the diagnostic workup of patients suspected of having IC[20]. Moreover, there are no specific urodynamic parameters that can predict a positive KCl test in patients with frequency-urgency syndrome or IC[21]. Urodynamics can be reserved for male patients, patients with associated urinary incontinence, refractory symptoms to standard therapy, and research. But the role of urodynamic testing is obvious in the differential diagnosis with other conditions.

- Uroflowmetry can indicate obstruction, dysfunctional voiding, and control for residual can indicate incomplete bladder emptying.
- Filling cystometry can objectivate detrusor overactivity. It permits us to evaluate bladder filling sensation, cystometric capacity. It can show if and at what volume pain and/or urgency occurs.
- The revised potassium test has shown prognostic value in bladder irrigation treatment[22].
- If video urodynamics are available, bladder and uretral diverticula, reflux, calculi, inflow in prostate, bladder neck, or other urethral pathologies can be diagnosed or excluded.

### Imaging

Imaging is needed if other pathology is suspected such as calculi, kidney pathology, malignancies, etc.

### Cystoscopy

- Cystoscopy under local anesthesia might be part of the workup to exclude other causes of CPPS.
- Cystoscopy under general anesthesia is mandatory when IC is suspected.

A proposal of a standardized technique has been given by Nordling et al.[6]. A rigid cystoscope that makes taking adequate biopsies more easy is used. Infusion liquid is positioned 80 cm above bladder level (which is often named hydrodistension). A dripping chamber allows us to notice when the inflow stops. Compression of the urethra around the cystoscope will prevent leakage. Maximum bladder capacity can be measured. Inspection includes observation of radiating vessels, coagulum or fibrin deposits, hyperemia, edema, scars, and other mucosal changes. When emptying the bladder, the degree of blood color of the fluid is evaluated. A second filling to one-third until two-thirds of the capacity permits inspection of the wall and biopsy. The classification of inspection as given by Nordling[6] is handy:

Grade 0: normal mucosa
Grade I: petechiae in at least two quadrants
Grade II: large submucosal bleeding
Grade III: diffuse mucosal bleeding
Grade IV: epithelial disruption, with or without bleeding/edema

A minimum of three biopsies is needed, taken at less than full bladder, including muscle, and from areas with clear pathological sights.

Short bladder distension proves to be a common practice, as recently discovered from a survey in the U.K.[23]. However, a marked variability in technique and little more than anecdotal evidence to support any particular approach was found.

The results of a pilot study of the feasibility of in-office bladder distension using electromotive administration of lidocaine (EMDA) was published recently[24]. Lidocaine EMDA proved superior to alkalized lidocaine as it allowed greater distension of the bladder for a longer time without eliminating the pain of bladder distension.

Some authors differ between a classical “ulcer type” disease and PBS, also an aspect in cystoscopy. The two conditions would differ in their histopathology, immunology, and neurobiology[25,26]. Classical ulcer disease displays reddened mucosal areas often associated with small vessels radiating towards a central scar, sometimes covered by a small clot or fibrin deposit[27]. The scar ruptures with the filling of the bladder, creating the characteristic “waterfall” type of bleeding. Classical ulcer disease and reduced bladder capacity under anesthesia are strongly interrelated. Nonulcer disease displays a normal epithelium at initial cystoscopy. The development of glomerulations after hydrodistension is a diagnostic sign lacking evidential authority.

Recently, it was shown that patients who met the NIDDK cystoscopic criteria had worse daytime frequency and nocturia and lower bladder capacity under anesthesia than those who did not meet the criteria. However, the two groups had similar urine markers and bladder biopsy findings, suggesting that cystoscopic criteria appear not to be able to identify a pathophysiological subset of patients with IC symptoms[28]. The cystoscopic findings of IC can also be found in patients without PBS and vice versa. Cystoscopic findings are, thus, not entirely pathognomonic for IC especially in the nonclassical IC. Cystoscopic findings in the classical IC subgroup of patients can be considered pathognomonic for the experienced investigator.

### Pathology Investigation

Not everyone is of the opinion that biopsies are mandatory for the diagnosis of IC. While in Europe a stringent clinical definition of IC has a strong emphasis on bladder biopsy, in the U.S., the trend to broaden the definition of IC was evident in the reports from the IC database study[29]: patients were accepted without biopsy.

An international consensus needs to be and will be developed. A number of fixation and staining methods to optimally utilize biopsies have been used. Optimal demonstration of these cells requires special fixation media (e.g., iso-osmotic formaldehyde acid [IFAA]) or, preferably, tryptase staining. Although not used in standard handling of biopsies, refinement of techniques no doubt increases diagnostic and scientific precision. Handling of the biopsies should be standardized and techniques have been proposed to do this[6].

Mast cell counting should be done in a standardized way and the pathology report should include several data as proposed before[6].

The case for pathological confirmation of IC is strongest for the classic disease as defined by ulcer or low anesthetic capacity. The ICI report[2] summarizes that the most commonly reported histological changes in classic IC included epithelial ulceration or denudation; submucosal inflammation; increase in lymphocytic infiltration; granulation tissue formation; edema; congestion; hemorrhage; detrusor fibrosis and myopathy; increased epithelial, submucosal, and detrusor mast cell number; and activation and increased neuronal staining.

Nonulcer IC is generally characterized by mucosal ruptures, submucosal hemorrhage, and mild inflammation in transurethral resection biopsies to normal histology in forceps biopsies in 50%[30,31]. This indicates that in nonulcer IC, forceps biopsy pathology is not very useful as a confirmatory test.

### IC and Associated Disorders

Several diseases and disorders have been shown to occur commonly in IC patients such as allergy, irritable bowel disease, Crohn’s disease, vulvodynia, fibromyalgia, migraine, asthma, and systemic autoimmune diseases such as lupus erythematosus and Sjögren’s syndrome[32]. A checklist to assess the possibility of an IC patient having such associated disorders is under development (first draft available through www.iicpn-foundation.org).

## REINVESTIGATION

One cannot stress enough the importance of accurate diagnostic exclusion of other pathologies before accepting the exclusion diagnosis of IC. Tissot et al. described the dangers of the recent trend to diagnose IC in a noninvasive way; 1% of the 600 patients they followed were found to have transitional cell carcinoma, 67% of whom had no hematuria[33].

The accuracy of the diagnosis and the value of each step of the diagnostic process were evaluated recently in a group of patients undergoing reinvestigation of IC[34]. The diagnosis of IC was confirmed only in 55%. Alternative diagnosis included recurrent urinary tract infections, detrusor overactivity, and vaginal candidiasis. Only one of five male patients had a confirmed diagnosis of IC. Previous studies have shown that IC diagnosis, if re-evaluated, will result in the diagnosis of other causes in a limited number of patients[35].

### CONCLUSION

Interstitial cystitis is a clinical diagnosis based on symptoms including pain in the bladder and/or pelvis. Its diagnosis is by exclusion of other diseases such as infection, malignancy, radiation, or drugs. A standardized protocol for this is not established so far, but international working groups make quick and important progress in this task.
